# Electromigration Forces on Atoms on Graphene Nanoribbons: The Role of Adsorbate–Surface Bonding

**DOI:** 10.1021/jacsau.3c00622

**Published:** 2023-12-18

**Authors:** Susanne Leitherer, Mads Brandbyge, Gemma C. Solomon

**Affiliations:** † Nano-Science Center and Department of Chemistry, University of Copenhagen, DK-2100 Copenhagen, Denmark; ‡ Department of Physics, Technical University of Denmark, DK-2800 Kongens Lyngby, Denmark; § Nano-Science Center and Department of Chemistry, Copenhagen University, DK-2100 Copenhagen, Denmark; ∥ NNF Quantum Computing Programme, Niels Bohr Institute, University of Copenhagen, DK-2100 Copenhagen, Denmark

**Keywords:** computational chemistry, electromigration, graphene nanoribbons, transition metals, electric fields

## Abstract

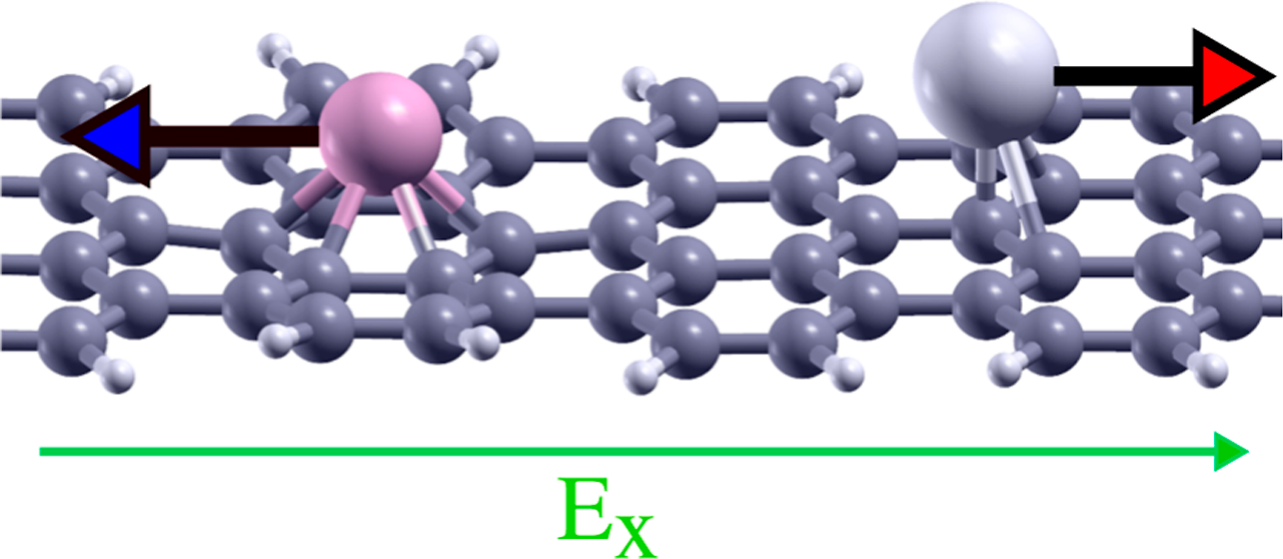

The synthesis of the two-dimensional (2D) material graphene and nanostructures derived from graphene has opened up an interdisciplinary field at the intersection of chemistry, physics, and materials science. In this field, it is an open question whether intuition derived from molecular or extended solid-state systems governs the physical properties of these materials. In this work, we study the electromigration force on atoms on 2D armchair graphene nanoribbons in an electric field using ab initio simulation techniques. Our findings show that the forces are related to the induced charges in the adsorbate–surface bonds rather than only to the induced atomic charges, and the left and right effective bond order can be used to predict the force direction. Focusing in particular on 3d transition metal atoms, we show how a simple model of a metal atom on benzene can explain the forces in an inorganic chemistry picture. This study demonstrates that atom migration on 2D surfaces in electric fields is governed by a picture that is different from the commonly used electrostatic description of a charged particle in an electric field as the underlying bonding and molecular orbital structure become relevant for the definition of electromigration forces. Accordingly extended models including the ligand field of the atoms might provide a better understanding of adsorbate diffusion on surfaces under nonequilibrium conditions.

## 1 Introduction

External control of adsorbate motion on surfaces is interesting both for the fundamental understanding of the mechanisms in play and for the development of novel nanoscale devices. Possible technical applications include, e.g., functional nanostructures designed by area-selective atom deposition,^1^ atomic/molecular switches,^2,3^ or ion traps based on electric fields, such as Paul traps,^4^ which are important for quantum computing.

In recent experiments, it has become feasible to place nanoparticles and even single atoms on surfaces and study their diffusion using atomic resolution scanning methods like scanning electron microscopy (SEM),^5^ scanning tunneling microscopy (STM), and atomic force microscopy (AFM).^6^ Besides mechanical manipulation by atomically sharp microscope tips, adsorbed particles can be moved by applying electrical fields, or, on conducting surfaces, by sending electrical currents through the structures, both causing electromigraton forces on the adsorbates.^7^ The electric field and current flow are achieved by either attaching metal electrodes or contacting the surfaces via a STM tip.

Two-dimensional (2D) graphitic surfaces like graphene,^5,8^ graphene nanoribbons (GNRs),^6^ and carbon nanotubes (CNTs)^9−11^ have recently gained interest for both experimental and theoretical studies of atom migration. Graphene-based materials exhibit a high stability and extraordinary, tunable electrical and thermal conductance properties,^12^ while their well-ordered 2D structure allows for an easy monitoring of surface migration processes. These 2D materials exist in the intermediate space between what we think of as molecular and what we think of as solid state. A large-scale graphene sheet might not resemble a molecule, but a narrow graphene nanoribbon certainly does. The question is therefore whether the intuition derived from either of these fields can be used to understand the behavior of 2D.

**Figure 1:**
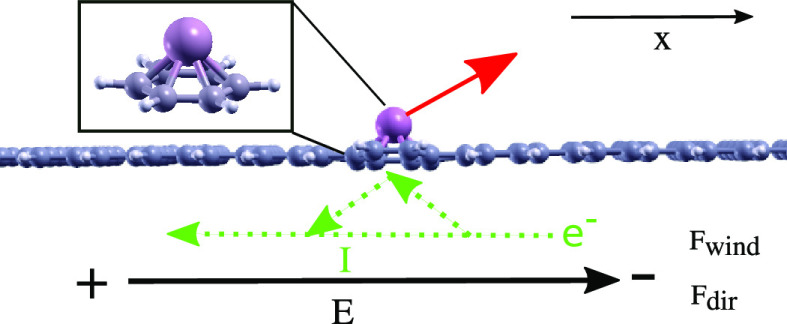
Single atom sitting on top of a 2D carbon nanostructure in an electric field, ***E***, applied along the surface direction (“transverse”) and under a current flow, ***I***. The atom experiences electromigration forces (red arrow), which consist of a contribution, ***F***_dir_, due to the electric field and a contribution, ***F***_wind_, due to scattering from electrons from the current. The inset shows the adatom and a benzene ring, representing a simplified model of the extended structure.

So far, different theoretical models for bias-induced atom diffusion on 2D surfaces have been employed to explain the experimental results. Thermally activated diffusion has been suggested for single Co atoms adsorbed on GNRs, subject to a conducting STM tip.^6^ In other works, bias-dependent adsorption energies and diffusion barriers were calculated.^11,13^ In general, the electromigration force is divided into a contribution from the electric field—the direct force—and the interaction with the current flow—the wind force^7^ (Figure 1). While the latter has been related to scattering of electrons by the adsorbate and the induced charge redistribution around the scatterer,^14−16^ the direct force has mainly been derived from the charge transfer between the adsorbate and surface and the induced adsorbate charge.^5,14,17^ Still, the understanding of the field-induced force is not extensive. While we have a classical understanding of metal atoms/clusters on surfaces in an electric field, where the force is related to the electric field via *F* = *qE*, with *q* being the atom charge, it is an open question as to whether this simple picture one would take from physics is enough to describe the behavior at the nanoscale.

In this work, we study the forces on single metal atoms placed on armchair graphene nanoribbons (aGNRs) in in-plane electric fields (Figure 1) using density functional theory (DFT). We project the problem onto a simple model of a metal (M) atom on benzene (C_6_H_6_ + M)^18^ and show how the molecular orbital structure is related to the induced forces. The problem does not look like traditional chemistry because of the bulk nature of the GNR + M systems. On the other hand, inorganic chemists have gained much knowledge about the bonding and interactions between metals and conjugated molecules using crystal field theory,^19,20^ where, e.g., ferrocene is a well-known example.^21^ The C_6_H_6_ + M model allows us to explain the results in an inorganic chemistry molecular picture, although the system is not “molecular”. We demonstrate how the chemical system can explain the results in the extended structure. Our results show that classical electrostatic models are not sufficient, as on the nanoscale, the electron distribution in the bonds and the molecular orbital/band structure become important.

For our study, we chose Co, Al, and Ag as adatoms because these particular elements were part of previous (experimental) studies,^5,6^ as well as due to their different adsorption sites and very different chemistry. To provide detailed insights into the relation between forces and molecular orbitals, we focus on 3d transition metal (TM) atoms. We know from inorganic chemistry that 3d TMs and their interaction with ligands can be very different, and we would expect to see a richer metal-specific behavior.

## 2 Results

### 2.1 Forces and Induced Atomic Charges

We start by calculating the induced forces on different atoms placed on armchair graphene nanoribbons (aGNRs) in the presence of a current and/or a transverse electric field. To understand the relation between the forces and the charge distribution, we compare the results to those of the induced atomic charges.

**Figure 2:**
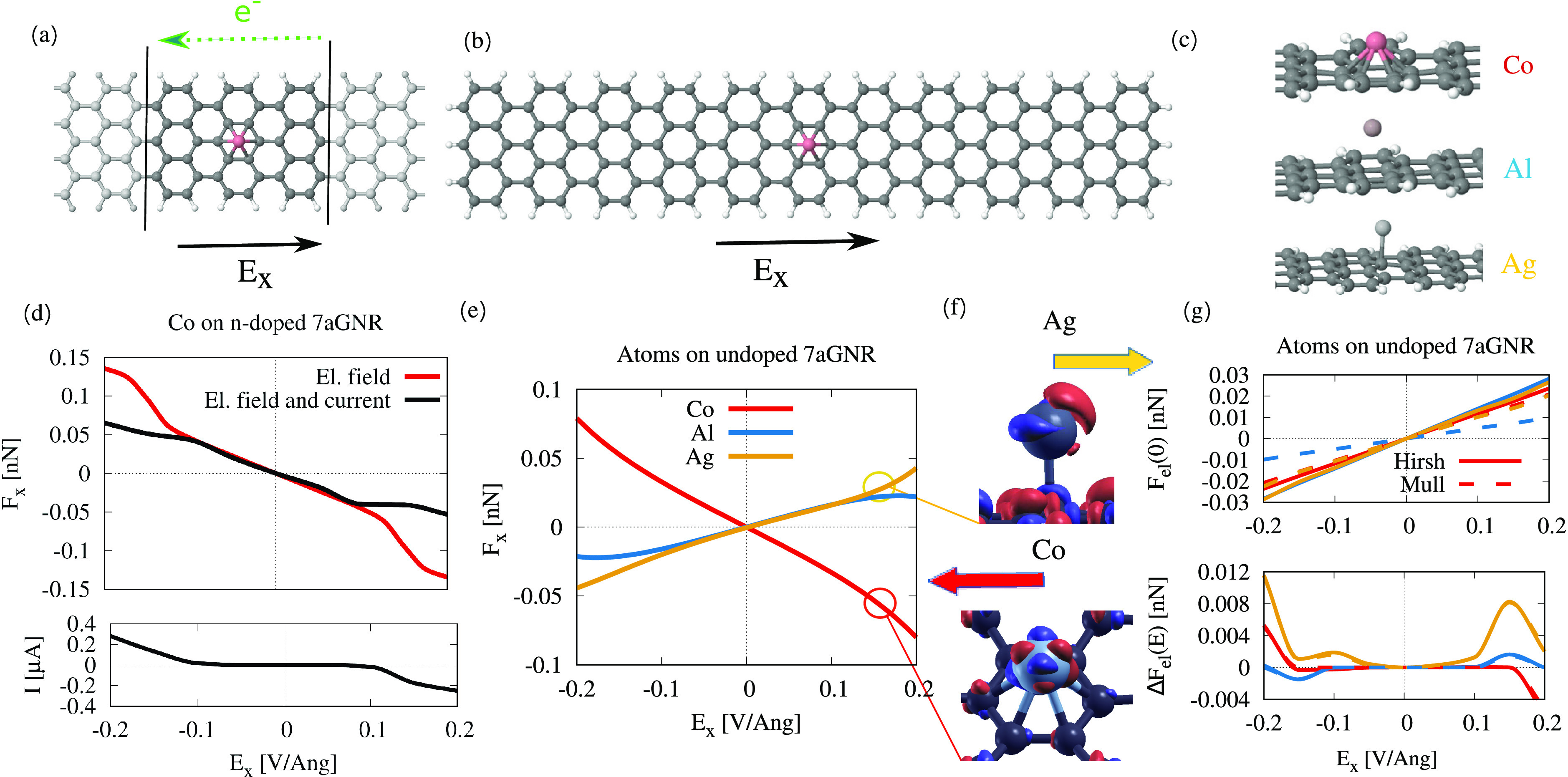
Single atoms adsorbed on a 7-aGNR in a transverse electric field/under finite bias experiencing electromigration forces. (a) Co atom on 7-aGNR with semi-infinite aGNR electrodes. A bias voltage is applied between the left and right electrode, which leads to an electric field and current flow. (b) Co atom on finite 7-aGNR in the transverse electric field *E*_*x*_. (c) Adsorption sites of single atoms (Co, Al, and Ag) on 7-aGNR: Co and Al prefer the hollow site, while Ag prefers the top site. (d) Top: Current- and field-induced force *F*_*x*_ on the Co atom (black line) from the transport setup shown in (a) and purely field-induced force (red line) from the setup shown in (b). Bottom: Current through system from (a) over an electric field. (e) Forces on Co, Al, and Ag atoms on 7-aGNR in an electric field. (f) Field-induced charge density on Ag and Co for *E*_*x*_ = 0.15 V/Å. (g) Top: Electrostatic forces *F*_el_(0) due to induced net charge *Q*(0) on Co, Al, and Ag upon adsorption on the GNR, obtained by Hirshfeld (solid) and Mulliken (dotted line) analysis. Bottom: Electrostatic forces Δ*F*_el_(*E*) due to field-induced charges Δ*Q*(*E*) over electric field *E*_*x*_.

The first systems we investigate are shown in Figure 2a,b: A single Co atom on a seven-atom-wide aGNR on the low energy “hollow” adsorption site, i.e., above the center of a six-membered ring in the GNR. In (a), semi-infinite seven-aGNR electrodes are attached to the left and right sides of the scattering region containing the adatom, between which a bias voltage can be applied. This results in a transverse, i.e., in-plane, electric field, *E*_*x*_, in the transport direction, *x*, and a current flow from one electrode to the other. In (b), we place a finite seven-atom aGNR in a transverse electric field, *E*_*x*_. This setup allows for the isolation of the direct force since no current flow is possible. To compare the two systems, we match the field strength, *E*_*x*_ (in V/Å), in the finite system to the bias voltage, *V*, in the extended structure with GNR electrodes by using *E*_*x*_ = *V*/*L*, where *L* is the ribbon length.

In both setups, we calculate the current/field-induced forces on the atoms using the TranSiesta software,^22,23^ where the force vector acting on atom *n* with coordinate  is given by force operator  via
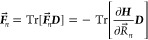
1with the Hamilton operator, ***H***, of the system and the corresponding density operator, ***D*** (see Section 4). We focus on the force on the adatom and its component *F*_*x*_ in the field direction.

Figure 2d shows a comparison of force *F*_*x*_ (top panel) on the Co atom in the presence of an electric field and a current [setup (a)] to force *F*_*x*_ on the atom in the same electric field but without current flow [setup (b)]. The current, shown in the bottom panel of Figure 2d, is negligible below field values of *E*_*x*_ ≈ 0.1 V/Å due to the GNR band gap. In this region, the forces are on the order of 0.05 nN and are the same in both setups since they are induced solely by the electric field. As soon as there is a current flow, we see a decrease of the induced total forces (black line), i.e., the current-induced force is opposite to the force from the electric field. In this region, the purely field-induced force is almost twice as high as the total force, which demonstrates the relevance of the field-induced force in our model.

Next, we compare different atoms on 7-aGNRs in an electric field: Co and Al on the hollow and Ag on the top site, i.e., on top of a C atom, as shown in Figure 2c, where the lowest energy site of the adatom depends on the atom type.^13,18,24^ The bridge site, i.e., on top of a bond, was not found to be a low-energy configuration for any of the metals studied here.

As shown in Figure 2e, the field-induced forces on the adatoms are linear up to a field strength of *E*_*x*_ ≈ 0.15 V/Å. The forces on Al and Ag are ∼0.02 nN at *E*_*x*_ = 0.1 V/Å and point in the field direction, while the forces on Co are slightly higher in magnitude and are directed opposite to the field.

We have analyzed the Hirshfeld atomic populations^25^ of the adatoms. From these populations, we estimate the force on the atom in the electric field from *F*_el_ = [*Q*(0) + Δ*Q*(*E*_*x*_)]·*E*_*x*_^′^, where *Q*(0) is the net charge of the adatom, i.e., the charge-neutral adatom transfers to the GNR at zero field, and Δ*Q*(*E*_*x*_) is the charge the electric field induces on the adatom. *E*_*x*_^′^ is the local electric field in the vicinity of the adatom, which we obtain from the slope of the electrostatic potential drop at the adatom. Due to screening from the environment, *E*_*x*_^′^ is slightly lower than the external electric field. As all atoms get positively charged upon adsorption on the GNR, the forces due to *Q*(0) (top panel of Figure 2g) point in the direction of the electric field. They are on the order of ∼0.02 nN at an electric field of *E*_*x*_ = 0.15 V/Å. The contribution to the direct forces calculated from Δ*Q*(*E*_*x*_) (bottom panel of Figure 2g) is significantly lower. A Mulliken analysis of the charges, which can depend significantly on the choice of basis set (for a more detailed explanation and comparison of the charge analysis methods, see ref (26)), revealed very similar trends [dotted line in (g)]. The electrostatic forces calculated from the net charge *Q*(0), with the contribution of Δ*Q*(*E*) being small, agree roughly in strength and, due to the positive sign of the net charge, in the direction with the DFT forces found for Ag and Al. However, the forces on Co do not match with the forces found in the DFT calculations, which point in the opposite direction and are considerably high. To visualize how the electric field polarizes the charge distribution, we show the field-induced charge density profiles in Figure 2f, where red indicates electron loss and blue indicates electron accumulation. For Ag, the induced density on the adatom is roughly forming a dipole, while for Co, the polarization of d-orbitals leads to a charge distribution of multipolar character. As these di- and multipoles are oriented along the field lines, they cannot explain the forces in field direction from the DFT calculation. We conclude that the field-induced forces cannot be reliably derived from the charges on the atoms.

### 2.2 Left and Right Bond Order

We next analyze the charge redistribution in the bonds between the adatom and surrounding C atoms. This allows us to connect the bond stability to transverse forces. We calculate an effective bond order similar to the Pauling bond order,^27,28^ which is defined as the difference between the number of electrons in bonding orbitals, *n*_B_, and the number of electrons in antibonding orbitals, *n*_A_, shared in a bond, divided by 2
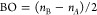
2Here, we calculate *n*_A_ and *n*_B_ from eq 7. We consider the field-induced change in bond order, ΔBO = BO(***E***) – BO(**0**), and define a left/right bond order as the sum of all BO contributions between the adatom and C atoms to the left/right.

**Figure 3:**
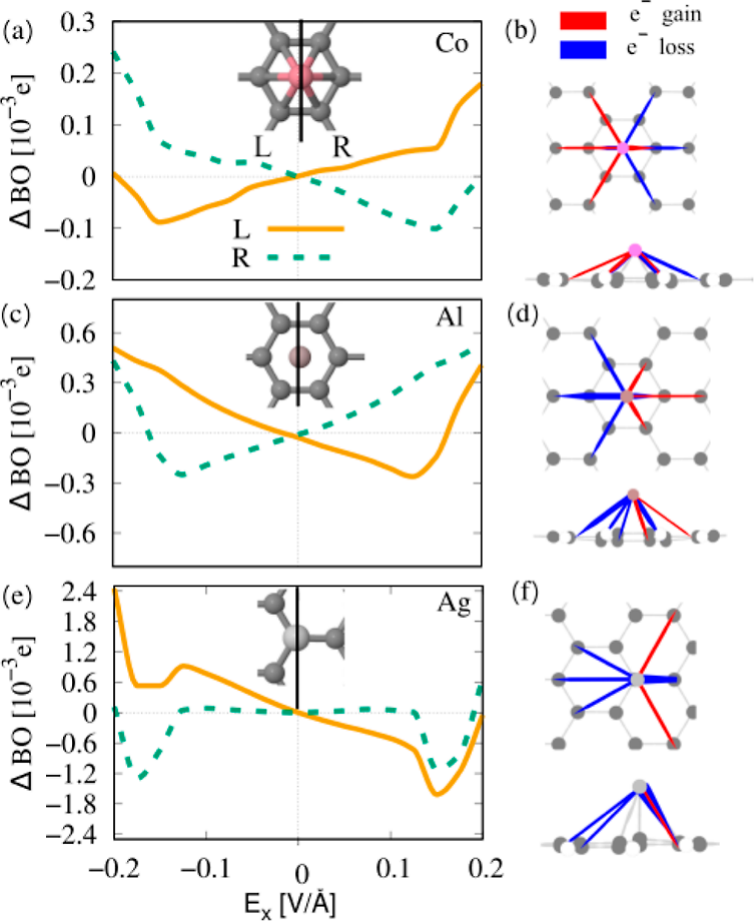
Analysis of the change in bond order (ΔBO) and the charge redistribution in the left and right bonds of Co, Al, and Ag on a finite 7-aGNR in a transverse electric field *E*_*x*_. (a) Left- and right field-dependent ΔBO of Co on 7-aGNR over electric field *E*_*x*_. The inset shows the partitioning into left and right bonds. (b) Visualization of induced bond charges (top and side view) for Co at *E*_*x*_ = 0.15 eV/Å, where blue depicts electron depletion and red accumulation. (c,d) Same analysis for Al on the hollow site and (e,f) for Ag on the top site. The charge redistribution in the bonds between atom and surface described by ΔBO predicts the direction of the field-induced force on the atom.

The field-induced left and right BO, ΔBO_L,R_, for Co, Al, and Ag is shown in Figure 3a,c,e. The inset shows partitioning into left and right bonds, respectively. For Co (a), at positive field values, the left BO increases, while the right BO decreases. Accordingly, bond weakening is induced on the right side, while the bonds on the left are strengthened. This results in a force on the atom to the left, i.e., a negative force. For negative fields, the left BO decreases, and the right BO increases, resulting in a force toward the right. At fields |*E*| ≤ 0.15 eV/Å, the left/right BO is linear and symmetric with respect to both the field and the left and right bonds. For high electric fields, the BO curves deviate from this linear and symmetric behavior, which we assign to charges induced in the not perfectly symmetric extended GNR and its edges influencing the local field near the atom bonds. In (b), the total gain and loss of electrons in the Co–C bonds are visualized for *E*_*x*_ = 0.15 eV/Å. Bonds, where the electron density increases as a function of the field, indicating bond strengthening, are shown in red, while bonds where electron density is depleted, inducing a bond-weakening, are shown in blue. We see that electron density increases mainly in bonds on the left, while it is depleted in bonds on the right, in accordance with ΔBO_L/R_ and the force directions.

For Al (Figure 3c), the right BO increases for positive field as electron density accumulates in the right bonds (d), while the left BO decreases, and electron density is depleted from the left bonds and vice versa for negative fields. The same principle holds for Ag (e,f); however, the BO is not symmetric with the field, as the top site is not mirror-symmetric with respect to a plane perpendicular to the long axis of the ribbon, as it would be, e.g., for a zigzag ribbon.

Our results show that the forces are related to the change in BO, i.e., the population of bonding/antibonding orbitals, with the BO separated into a left and right contribution determining the force direction. Specifically, the change in BO is caused by polarization of the charge density in the bonds in the electric field. To explain the magnitude of the force, extended models must be employed. We know that the bond population can be taken as a measure for the force in bond direction, i.e., the bond force.^16^ The component in the *x*-direction, *F*_*x*_, will then depend not only on the magnitude of the left and right BO but will also be scaled by the adsorption height of the atom. For example, Co (1.25 Å) sits much closer to the surface than Al (2.06 Å), resulting in a larger component, *F*_*x*_, for Co due to the shorter bonds. Also, the bond type plays a role. For Co, the main contribution to the bond charge comes from the π bond between its *dxz* orbital (see the discussion later) and the GNR π orbital, while for Al, the p_*z*_ orbital binds to the GNR π system. The former has a larger *x*-component in comparison to the latter. This explains why ΔBO of Co is a factor of 2 smaller than ΔBO of Al, while the resulting forces on Co are higher than those on Al, suggesting a material-specific relation between ΔBO and the magnitude of the force.

### 2.3 Forces and Molecular Orbital Structure

In the next step, we will focus on Co as an example and compare it to other 3d TM atoms. As a simple model, we consider a single atom adsorbed on benzene (C_6_H_6_ + M) to provide detailed insights into how the forces and bond charges are related to the molecular orbital (MO) structure. We will later show how the orbitals of C_6_H_6_ + M can explain the forces found in the extended GNR structures. The C_6_H_6_ + M model has been successfully employed to understand properties like adsorption energies of TM metals on graphene and CNT structures in other works^18,29^ and is a convenient model for atoms on the hollow site of hexagonal graphene-like surfaces. We will here adopt the notation from previous works for the molecular orbitals according to their symmetry properties. Furthermore, we utilize the density of states (DOS) and crystal orbital overlap population (COOP)^30^ (eqs 4 and 6 in Section 4) to analyze the force contributions in terms of the molecular orbital structure, a method that was previously used for a number of different nanoscale systems.^15,16,31,32^ The results presented in the following do not include spin polarization, which often plays a role in systems with TM atoms. We have performed spin-polarized test calculations and found the same force order of magnitude and trends (SI).

**Figure 4:**
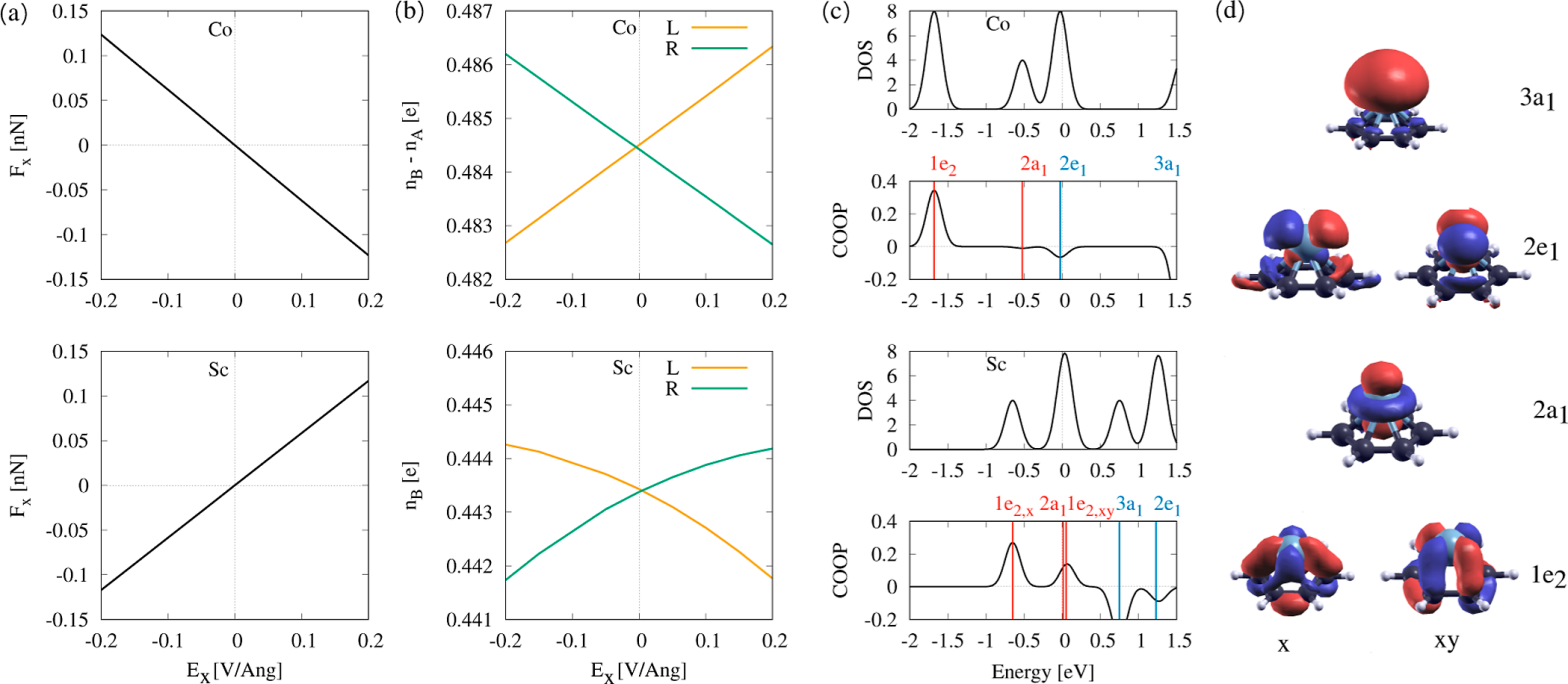
Comparison of electromigration forces, bonding and antibonding electrons, and energy states for Co and Sc on benzene (“C_6_H_6_ + M” model) in an electric field *E*_*x*_. (a) Field-induced force *F*_*x*_ on Co and Sc on benzene. (b) Field-induced bonding and antibonding electrons, *n*_B_ and *n*_A_, on the left- and right-hand side of the adatoms. While for C_6_H_6_ + Sc, only bonding orbitals are occupied (*n*_A_ = 0), C_6_H_6_ + Co exhibits electron density from antibonding orbitals 2*e*_1_. (c) Density of states (DOS) of C_6_H_6_ + M and crystal orbital overlap population (COOP) between the adatom and C_6_H_6_ for Co (top) and Sc (bottom). In the COOP, the energetic positions of the C_6_H_6_ + M states is shown. The COOP indicates the nature of the states (positive = bonding and negative = antibonding). (d) C_6_H_6_ + M molecular orbitals denoted according to their symmetry.^18^

In Figure 4a,b, we show the forces along with the left and right numbers of bonding/antibonding electrons *n*_B/A_ for two TMs on C_6_H_6_: Co (top) and Sc (bottom). The forces on Co [(a), top] point against the field, while for Sc [(a), bottom], they are oriented with the field direction. Accordingly, the number of left and right bonding electrons increases/decreases (b). Note that Co has antibonding electrons, while Sc has only bonding electrons.

Next, we analyze how the energetic states contribute to *n*_A_ and *n*_B_. To this end, in (c), the DOS and corresponding COOP are shown. In the DOS and COOP, the energetic position of the C_6_H_6_ + M molecular orbitals, depicted in (d), are shown. The sign of the COOP indicates whether a state has bonding (positive) or antibonding (negative) character. We find that the bonding states 1e_2_ and the nonbonding 2a_1_ are occupied for both Co and Sc, with 1e_2_ being quasi-degenerate and significantly lower in energy for Co. The antibonding 2e_1_ states are occupied only for Co, while they are empty for Sc.

The integration of the COOP over energy yields the number of bonding/antibonding electrons, *n*_A_ and *n*_B_ (cf. eq 7 in Section 4), from which we derive the BO. By calculating the COOP separately for the left and right bonds at zero and finite field, we obtain ΔBO_L/R_ and can compare whether ΔBO is higher in the left or in the right bonds. By comparing the cumulatively integrated COOP over energy, we can estimate the contribution of the different energy states to ΔBO_L/R_.

The integration of the COOP for C_6_H_6_ + Co is demonstrated in the SI. We find that the main contribution to ΔBO_*L*/*R*_ comes from state 2*e*_1_(*x*), while the contributions of the lower states cancel each other. The direction of the force on Co is therefore related to the occupation of state 2*e*_1_(*x*).

**Figure 5:**
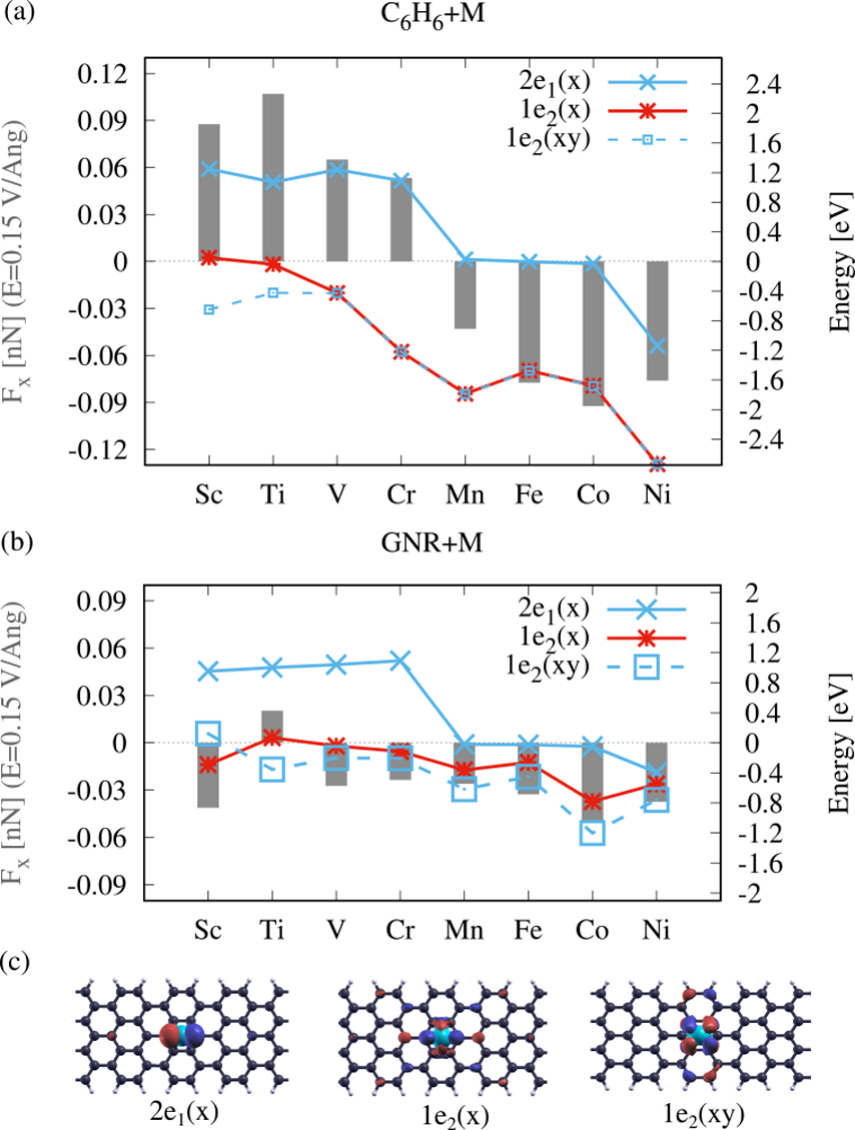
Correlation between the electromigration force and energy states close to *E*_F_ = 0 eV. (a) Force *F*_*x*_ at field *E*_*x*_ = 0.15 V/Å on adatoms (gray bars) and energetic position of state 2*e*_1_(*x*) (blue), 1*e*_2_(*x*) (red), and 1*e*_2_(*xy*) (blue dashed) for atoms on benzene (C_6_H_6_ + M) and (b) on 7-aGNRs (GNR + M). (c) Molecular orbitals 2*e*_1_(*x*), 1*e*_2_(*x*), and 1*e*_2_(*xy*) of Co on 7-aGNR. The forces on the early TMs are related to the populations of the 1*e*_2_ states, while the late TMs are dominated by state 2*e*_1_(*x*), being closer to *E*_F_ for both C_6_H_6_ + M and GNR + M.

We have performed the same analysis for eight 3d TMs (Sc, Ti, V, Cr, Mn, Co, Fe, and Ni) on C_6_H_6_ and on 7-aGNR (cf. Figure 5). In the following, we will show how the field-induced forces on these adatoms can be understood in terms of the individual contributions of their molecular orbitals to ΔBO_L/R_. We find that the energetic states that are closest to the Fermi energy give the dominant contribution to ΔBO, as contributions from lower states cancel one another. Thus, we can conclude the force direction from the states close to E_F_.

In Figure 5a, we compare the energetic position of the relevant states 2*e*_1_(*x*), 1*e*_2_(*x*), and 1*e*_2_(*xy*) to the induced forces at *E*_*x*_ = 0.15 V/Å, shown as gray bars, for C_6_H_6_ + M. The energies of the states are shown in colors corresponding to their contribution to the left/right bond order, i.e., their influence on the force direction: blue for ΔBO_L_ > ΔBO_R_ (negative force) and red for ΔBO_L_ < ΔBO_R_ (positive force). Note that the MOs roughly keep this field dependence for all TMs, while their energetic position relative to E_*F*_ changes.

For the early TM atoms (Sc–Cr), state 2*e*_1_(*x*) is unoccupied and far from the Fermi energy. The dominant contribution comes from state 1*e*_2_(*x*) with ΔBO_L_ < ΔBO_R_, resulting in a force from left to right, i.e., a positive force. State 1*e*_2_(*xy*) has only small negative contributions to the force (ΔBO_L_ > ΔBO_R_). For the late TM atoms (Mn–Ni), state 2*e*_1_(*x*) gets shifted close to/below the Fermi energy, and the forces become negative, as soon as state 2*e*_1_(*x*) with ΔBO_L_ > ΔBO_R_ is occupied.

In (b), the comparison of the MO energies to the induced forces at *E*_*x*_ = 0.15 V/Å is presented for the same TM atoms adsorbed on 7-aGNR. As a general difference to the C_6_H_6_ + M model, the MOs in the 7-aGNR systems are more broadened due to the coupling to more extended GNR states (Figure 5c). In (b), we indicate this by a larger marker size of the energy state curves. For the late TMs (Mn–Ni), the forces we find are similar to those in the C_6_H_6_ + M model. Here, 2*e*_1_(*x*) remains the dominant state at the Fermi energy, leading to negative forces. The 1*e*_2_ states are shifted up in energy, and their degeneracy is lifted. For the early TMs (Sc–Cr), we find larger deviations between the 7-aGNR + M and the C_6_H_6_ + M model. The forces on the atoms on the GNRs, except for Ti, are now negative and lower in magnitude. While state 2*e*_1_(*x*) remains unoccupied and far away from the Fermi energy, the contributions of the 1*e*_2_ states compete. For Sc, V, and Cr, state 1*e*_2_(*xy*), with negative force contributions, which was not dominant in the C_6_H_6_ + M system, is now strongly broadened and leads to a negative force. For Ti, 1*e*_2_(*x*) remains the dominant state, resulting in a positive force.

### 2.4 Influence of Doping and Adatom Site

So far, we have focused on undoped GNRs and TM atoms on the hollow site. In the last section, we show how significantly the field-induced forces depend on the doping levels and adsorption sites, as those have an influence on the MO structure and energies, which we demonstrate for Co on 7-aGNR.

**Figure 6:**
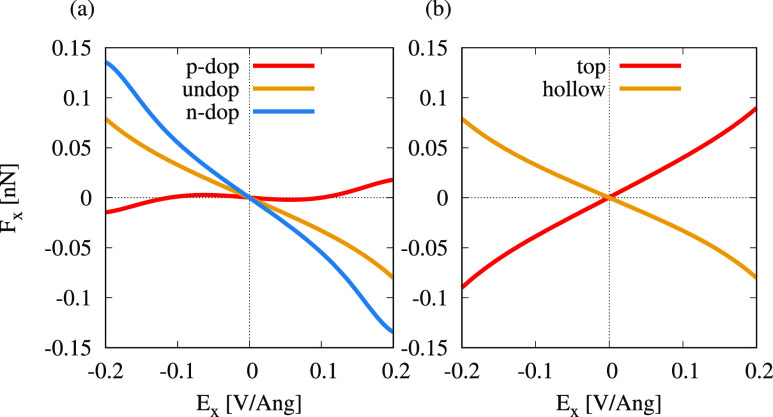
Electromigration forces on a Co atom on 7-aGNR in a transverse electric field, *E*_*x*_, depending on the doping level and atom site. (a) Force *F*_*x*_ over electric field *E*_*x*_ on a Co atom on a n-, p-, and undoped 7-aGNR. (b) Comparison of force *F*_*x*_ over electric field *E*_*x*_ on Co on top and hollow site of an undoped 7-aGNR.

As shown in Figure 6a, the forces on the adatom are oriented against the field for undoped and n-doped 7-aGNRs. The largest forces are found for Co on n-doped 7-aGNR, as n-doping leads to a higher electron density and thus higher bond populations in the system. P-doping shifts the relevant states out of the occupied energy range. Therefore, the forces vanish for low fields and are significantly smaller for high field, pointing in field direction. Similarly, the forces change direction when Co is on the top site (Figure 6b). Here, the ligands are very different, yielding a MO structure very different from the one shown in Figure 4d.

## 3 Conclusions

Summing up, we have presented an ab initio study of the forces that act on single atoms adsorbed on 7-aGNRs in a transverse electric field. For Co and other 3d TM adatoms, we found non-negligible field-induced forces, which for high fields are directed against the wind force. The induced charge distribution on the adatom was not sufficient to explain the direct forces. Instead, the effective left and right BO (eq 2), describing the electron population in the bonds between the adatoms and the surface, can explain the force directions. We have demonstrated that for Al and Ag adatoms, where the adsorbate charge gives a better estimate of the direct force, this principle is also valid.

Utilizing a simplified molecular model including the adatom and only one benzene ring, we found that the change in BO is due to the change in bond charge of only a few molecular orbitals in the proximity of the Fermi energy. We have demonstrated for 3d TMs that the MOs of C_6_H_6_ + M can, with small deviations, be generalized to understand the results of the extended GNR models.

Our calculations are relevant in the low current regime and illustrate how the electronic redistribution in the bonds, rather than the direct interaction with the adsorbate charge, is important. It should be noted that the induced forces do not necessarily lead to displacement of the adatom due to the presence of a diffusion barrier. In the experiments by Preis et al.,^6^ the heating due to the current passing through the Co-nanoribbon system into the Au(111) substrate is responsible for the nondirective Co motion. On the other hand, for strong currents, for small gap GNR or graphene, the electronic resonance structure of the adsorbate can dominate the picture as shown in a recent study by Choi and Cohen.^14^

The magnitude of the forces we found are in the order of 0.05–1 nN for fields of 0.15 V/Å, which is an order of magnitude lower than bond breaking forces reported, e.g., for Au–Au nanocontacts,^33^ but might still be relevant in experiments. While the forces we found for metals like Al agree in magnitude and direction with previous results,^5^ it will be interesting to see if the results presented for TM atoms such as Co are reproducible in experiments.

In conclusion, we have demonstrated several systems of atoms on surfaces in electric fields where classical models for the electrostatic forces were not satisfactory, revealing forces that were unambiguous in their direction relative to the field. Extended models are needed that consider the ligands of the adatom and its MO structure. The field-dependent BO gives insights into how bonds between the adsorbate and the surface are influenced by the field and can, with improvements, provide a new understanding of electromigration forces on atoms on the nanoscale.

## 4 Method

We have performed electronic structure calculations based on DFT and (nonequilibrium) Green’s functions (NEGF) as implemented in TranSiesta.^22,23^ The physical quantities presented here were analyzed using postprocessing tools implemented in sisl.^34^ The calculations were performed using a 300 Ry mesh cutoff, double-ζ polarized basis sets, the PBE + GGA exchange–correlation functional,^35^ and otherwise default parameters. To introduce doping, we apply the field-effect gate model of ref (36), where a charged plane is placed underneath the GNR. The transport structures, finite GNR, and benzene structures were relaxed at zero bias/field with a force tolerance of 0.005 eV/Å. Using these relaxed geometries (which can be found here), a finite bias voltage/electric field was applied, and the electromigration forces and charge densities in the Born–Oppenheimer approximation were calculated. To obtain the voltage/field-induced forces and charges, we subtract the zero voltage/field quantities from those at a finite voltage/field.

The electronic part of the force acting on atom *n* with coordinate  is given by eq 1. For finite bias calculations with semi-infinite electrodes, the nonequilibrium density matrix of the device region can be written as
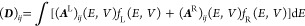
3where *f*_L/R_ are Fermi distributions describing the electron population in the left and right electrodes. The left and right spectral densities, ***A***^L^, ***A***^R^, are derived from the left and right going scattering states in the device region.^22^

For finite systems in an electric field, a ‘molecular’ density of states is defined using
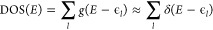
4where ϵ_*l*_ are the molecular eigenenergies and as distribution *g* we chose a Gaussian with a smearing of 0.1 eV. The density matrix is given, with the molecular orbitals ψ_*l*_ = ∑_*k*_*c*_*lk*_ϕ_*k*_ by
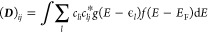
5with *f* being the Fermi distribution.

As a measure for the force direction, we define a left and right effective bond order BO = (*n*_B_ – *n*_A_)/2 from the bond populations of bonding and antibonding orbitals, *n*_B_ and *n*_A_, which we also term bonding and antibonding electrons, respectively. These are derived from the COOP^30^
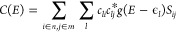
6where we sum over atomic orbitals (*i*, *j*) belonging to the atoms *n*, *m*. The number of bonding/antibonding electrons are then obtained from
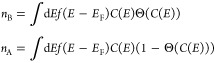
7

## Supporting Information Available

Field-induced forces on Co on 7-aGNR in *y* and *z* direction, spin-polarized calculation of forces, Co on coronene (C_12_H_24_), detailed COOP analysis for Co and Sc on C_6_H_6_ and 7-aGNR, and Co on 11-aGNR (PDF)
