# Supplementary material for: Electromigration Forces on Atoms on Graphene Nanoribbons: The Role of Adsorbate–Surface Bonding
Source: JACS Au. 2023 Dec 18;4(1):189–96. doi: 10.1021/jacsau.3c00622 (PMC10806770; doi:10.1021/jacsau.3c00622)
Supplement: Supplementary file 1 — au3c00622_si_001.pdf [file au3c00622_si_001.pdf]

Supplemenatry Information for 'Electromigration forces on atoms on graphene nanoribbons: The role of adsorbate-surface bonding'

Susanne Leitherer,† Mads Brandbyge,‡ and Gemma C. Solomon*,¶,§

†*Nano-Science Center and Department of Chemistry, University of Copenhagen, DK-2100 Copenhagen, Denmark*

‡*Department of Physics, Technical University of Denmark, DK-2800 Kongens Lyngby, Denmark*

¶*Nano-Science Center and Department of Chemistry, Copenhagen University, DK-2100 Copenhagen, Denmark*

§*NNF Quantum Computing Programme, Niels Bohr Institute, University of Copenhagen, DK-2100 Copenhagen, Denmark.*

E-mail: gsolomon@chem.ku.dk

1 Forces in x,y and z direction

In the manuscript, we evaluate and compare the force component F_x in the direction of the electric field. In Fig. 1, all three components are shown for the adatoms Co, Ag and Al on 7-aGNRS, including F_y lateral to the ribbon and F_z perpendicular to the ribbon surface. While the out-of-plane force component F_z on the Co atom (a) is very small compared to the transverse component F_x, Al (b) and Ag (c) do also exhibit a non-negligible component F_z, pushing the atom away from the GNR surface. F_y is zero for all adatoms.

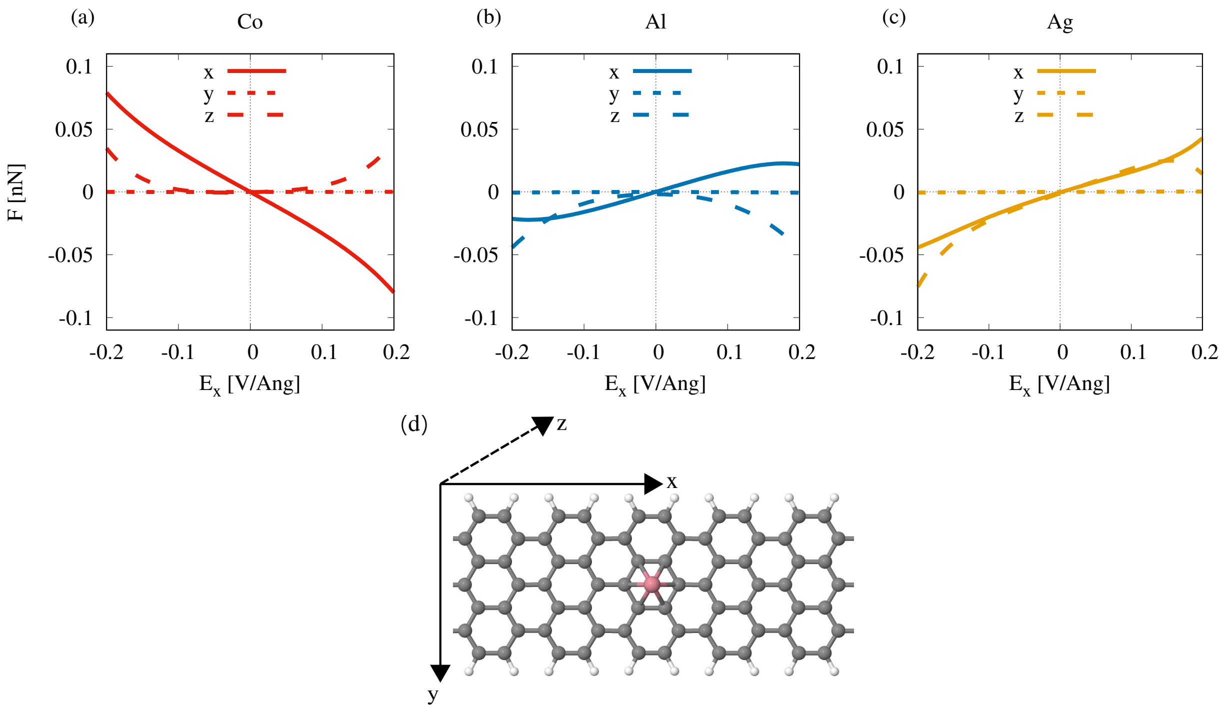

Figure 1: (a-c) Force components in x, y and z direction for Co, Al and Ag atoms on an undoped 7-aGNR over electric field in x-direction,(d) Coordinate system of the GNR+metal structure: x refers to the direction along the GNR, while z is the out-of-plane component.

2 Spin-polarized calculations

We have perfomed spin-polarized calculations in TranSiesta[1,2] for the adatoms Co, Fe and Ni on benzene and on 7-aGNRs in electric fields (Fig. 2). An extended study of the magnetic properties of 3d TM atoms on graphene/C_6H_6 is presented in Ref. 3.

For Ni, which has spin moment 0, we see no difference between the spin polarized and unpolarized calculations, for the atom placed on benzene as well as on the GNR.

The difference in the forces is in the order of ±0.015 nN for Co (with spin moment 1) and ±0.025 nN Fe (with spin moment 2). We conclude that spin polarization has an influence on the force magnitude, however it does not revert force directions, or greatly change the order of magnitude and trends presented in this work.

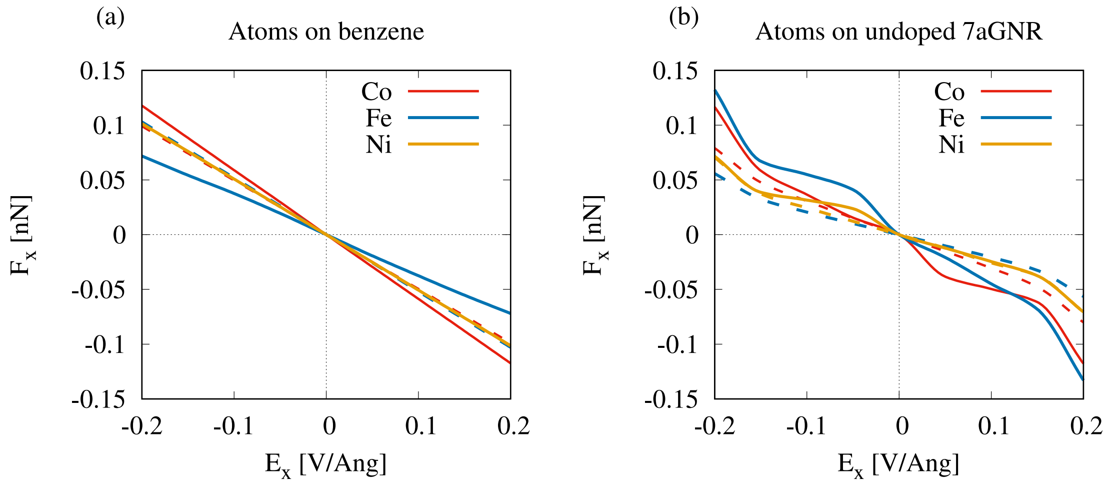

Figure 2: Forces on Co, Fe and Ni with (solid) and without (dashed line) spin polarization, on benzene (a) and on a 7-aGNR (b). Spin moment on Benzene: Co:1 , Fe:2, Ni: 0

3 Co on $C_{24}H_{12}$

To demonstrate that the simplified model discussed in the manuscript can be extended to a larger system with the same conclusion still being valid, we have considered atoms on coronene($C_{24}H_{12}$). In Fig. 3, the forces and field-induced change in bond order for a Co adatom on coronene (see inset in (a)) are shown. The forces and induced bond order, ΔBO are very similar to those in the C_6H_6+Co model, and slightly smaller in the $C_{24}H_{12}$+Co model, which might be due to screening and differences in the geometric structure.

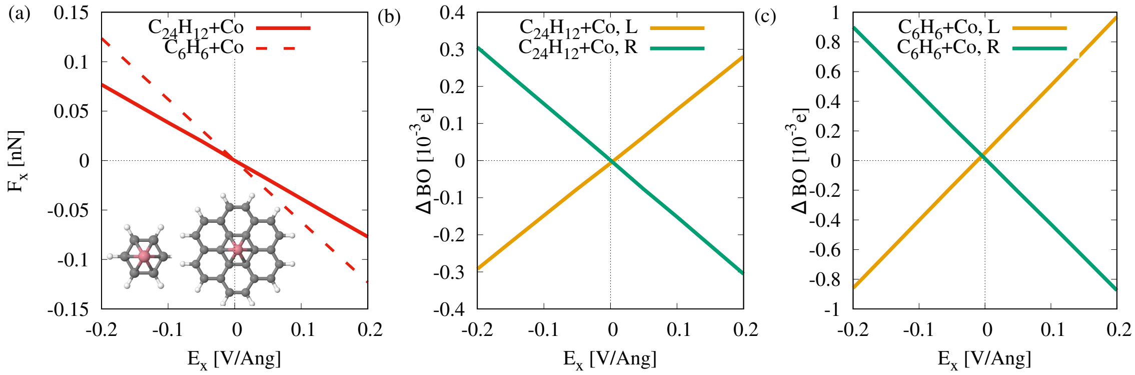

Figure 3: Comparison of $C_{24}H_{12}$+Co and C_6H_6+Co. (a) Forces F_x over electric field E_x. (b) Change in left and right BO in $C_{24}H_{12}$+Co and (c) in C_6H_6+Co.

4 COOP analysis for 3d TMs on C_6H_6 and on 7-aGNRs

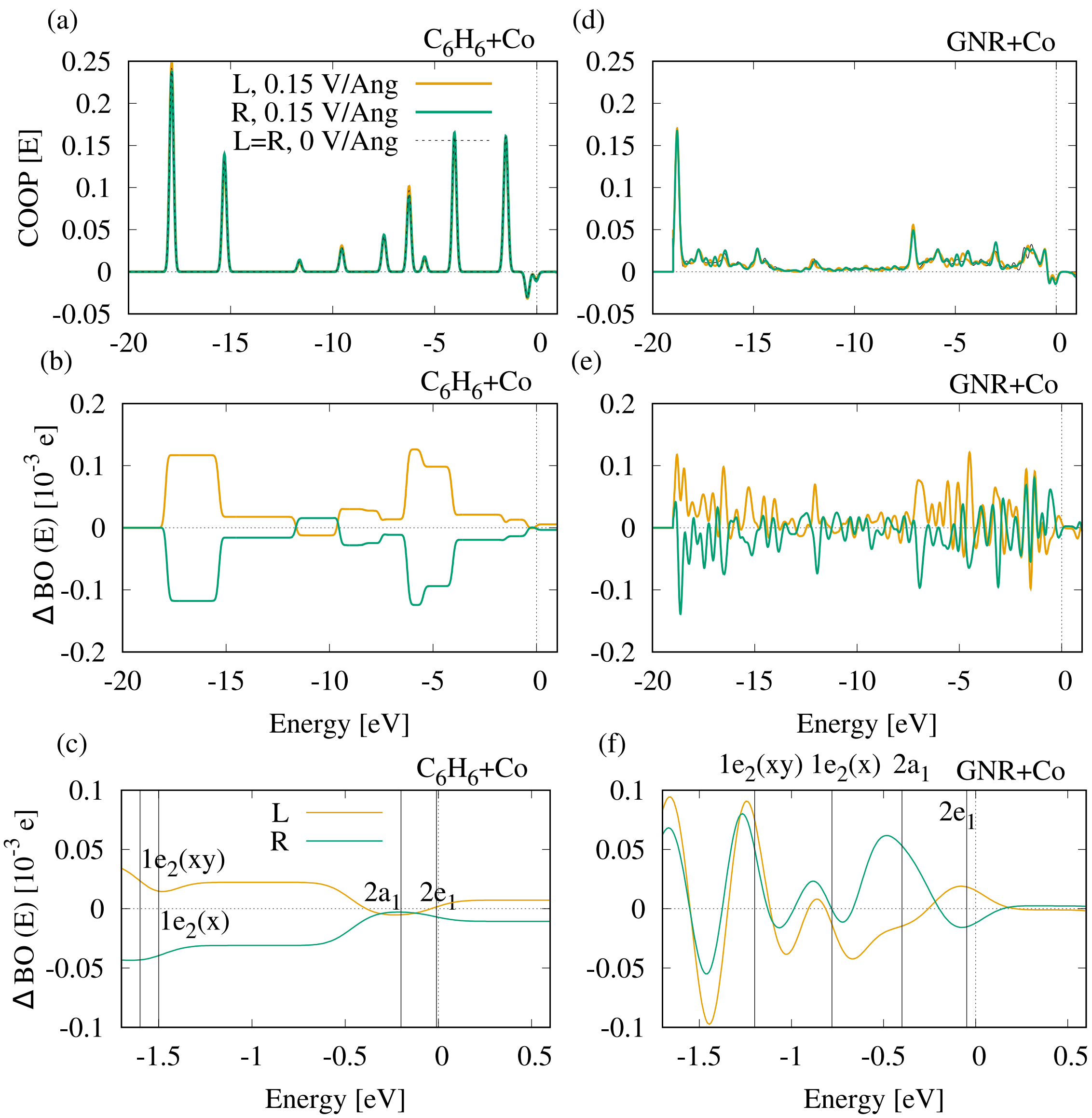

Figure 4: COOP at $E_x = 0.0$ V/Å and $E_x = 0.15$ V/Å for left and right Co-C bonds, and cumulative induced bond order ΔBO at $E_x = 0.15$ V/Å over energy. (a) COOP, (b) cumulative ΔBO and (c) zoom into energy range close to E_F for ΔBO of C_6H_6+Co (left) and of 7aGNR+Co (right). (d-f) The same analysis for C_6H_6+Sc (left) and of 7aGNR+Sc (right).

In the manuscript we have shown that the effective bond order (BO) is obtained from

$$\text{BO} = (n_B - n_A)/2 = \frac{1}{2}\int_{-\infty}^{E_F} \mathrm{d}E\ \mathrm{C}(E), \tag{1}$$

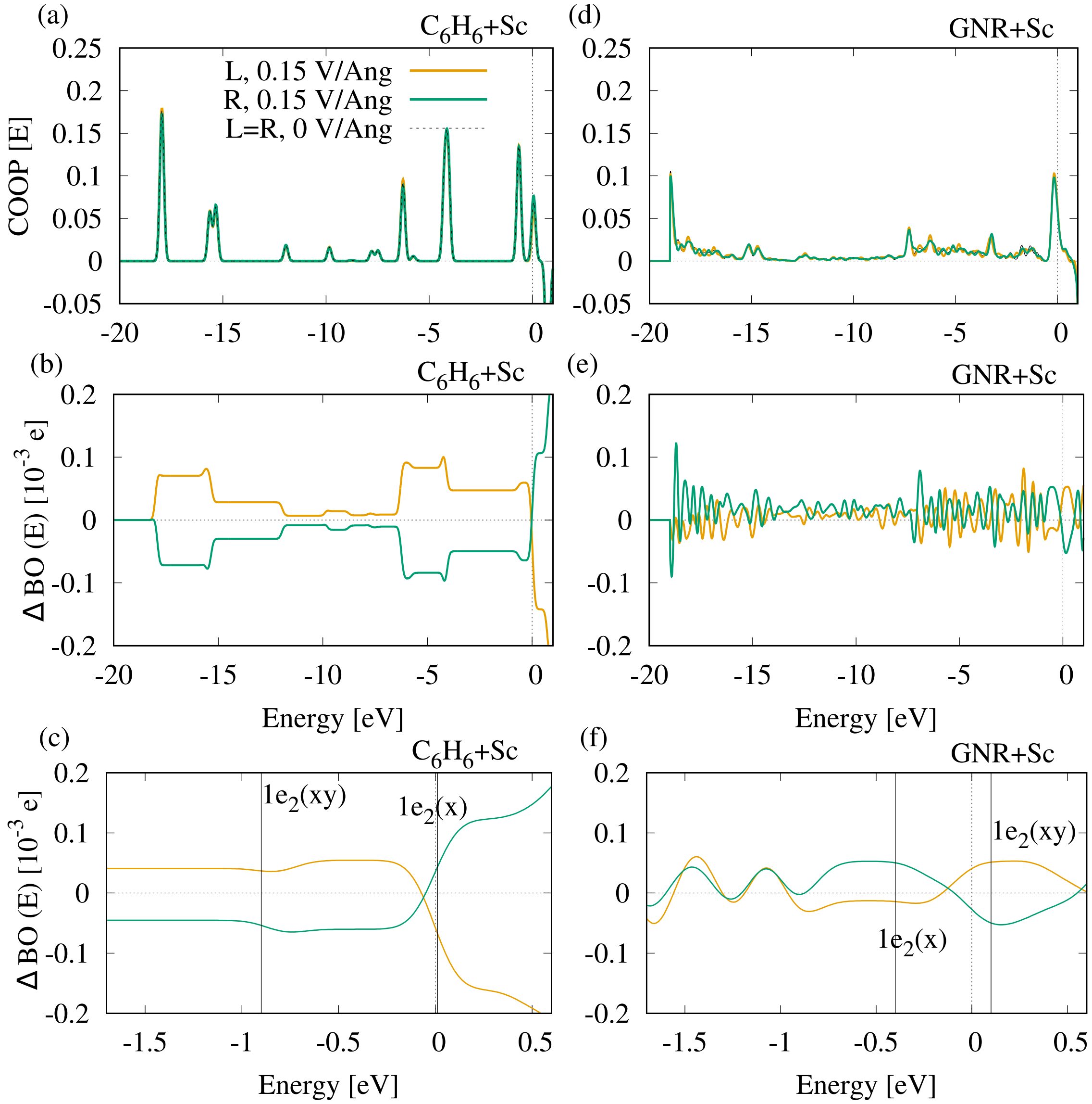

Figure 5: COOP at $E_x = 0.0$ V/Å and $E_x = 0.15$ V/Å for left and right Sc-C bonds, and cumulative induced bond order ΔBO at $E_x = 0.15$ V/Å over energy. (a) COOP, (b) cumulative ΔBO and (c) zoom into energy range close to E_F for ΔBO of C_6H_6+Co (left) and of 7aGNR+Co (right). (d-f) The same analysis for C_6H_6+Sc (left) and of 7aGNR+Sc (right).

where n_A, n_B are the number of bond electrons in bonding/antibonding orbitals, which can be obtained from integrating the energy-dependent crystal orbital overlap population (COOP).[4] In our calculations, the Fermi energy is at $E_F = 0$. The field-induced BO is obtained from ΔBO= BO(E_x)-BO(0), and calculated separately for bonds on the left and right hand side of the adatom.

In order to understand the contributions of the individual energy states to $\Delta\mathrm{BO}_{L,R}$, we analyze the left and right COOP cumulatively integrated over energy. This is demonstrated for the adatoms Co and Sc in Fig. 6 and Fig. 5, respectively .

In Fig. 6 (a), we compare the left and right COOP for Co on benzene for $E_x = 0$ V/Å to the COOP at $E_x = 0.15$ V/Å. We see that the states are only slightly shifted by the field and deformed leading to different peak width and heights. In (b), by integrating the COOP over all states (energy range from -20:0 eV) and subtracting the zero-field COOP, we analyze the amount of field-induced $\Delta\mathrm{BO}_{L,R}$ that is accumulated over all respective energies. We see that there are contribution to $\Delta\mathrm{BO}_{L,R}$ at lower energies, which add up to zero, so that only the contributions of the state $2e_1(x)$ at 0.04 eV is relevant (see zoom into energy range aroung E_F in (c)). This state has a higher (lower) bond charge for the left (right) bonds, explaining the negative force, i.e. a force which is pointing towards the left. For Co on a 7-aGNR, the same analysis is shown in Fig. 6 (d-f). The contributions of state state $2e_1$ leads to $\Delta\mathrm{BO}_L > \Delta\mathrm{BO}_R$. Note that for the quasi-degenerate state $2e_1(xy)$ no significant $\Delta\mathrm{BO}$ is induced, as can also be shown by a bond-resolved analysis of the COOP.

Fig. 5 shows the analysis for Sc on benzene and on a 7-aGNR in a transverse electric field of $E_x = 0.15$ V/Å. Again, the contribution to $\Delta\mathrm{BO}_{L,R}$ from states at lower energies cancel out and the main contribution is from states near E_F. The relevant contribution for Sc on Benzene come from state $1e_2(x)$ with $\Delta\mathrm{BO}_R > \Delta\mathrm{BO}_L$ (Fig. 5 (b,c)), leading to a positive force. For Sc on the ribbon (d-f), state $1e_2(xy)$ is closer to E_F and contributes $\Delta\mathrm{BO}_L > \Delta\mathrm{BO}_R$, which results in a negative force.

5 Atoms on a 11-aGNR

We have performed test calculations of atoms placed on broader graphene nanoribbons, as on wider ribbons the symmetry, especially in y-direction, is more similar to the C_6H_6+M symmetry. We compare the forces on Co and Sc placed on Benzene, a 7-aGNR and a 11-

aGNR in Fig. 6. For Co (a), we find similar forces in all three models. For Sc (b), we had found that the forces in the 7-aGNR+M model are opposite to the forces in the C_6H_6+M model. For 11-aGNRs, the sign of the forces is now the same as in the Benzene model. We conclude that the C_6H_6+M model might better match the results of broader ribbons.

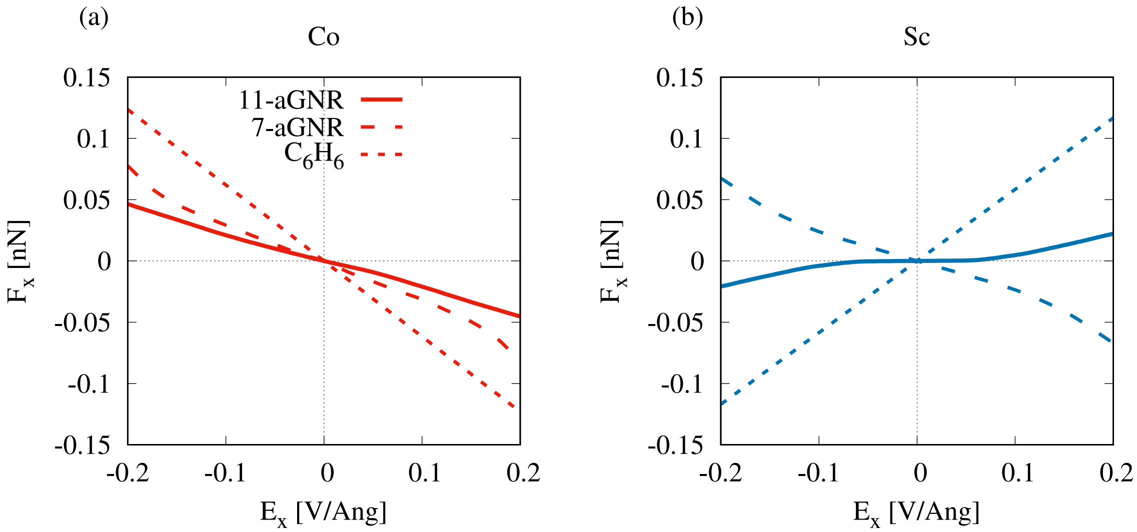

Figure 6: Field-indued forces acting on a Co (a) and Sc (b) atom placed in the central hollow position on a 11-aGNR, a 7-aGNR and Benzene. While the forces on Co are comparable in all three structures, the forces on Sc on the broader 11-aGNR are more similiar to those in the Benzene model, as the symmetry is slightly broken in the 7-aGNR case.

6 References

References

(1) Brandbyge, M.; Mozos, J.; Ordejon, P.; Taylor, J.; Stokbro, K. Density-functional method for nonequilibrium electron transport. *PHYSICAL REVIEW B* **2002**, *65*, 165401.

(2) Papior, N.; Lorente, N.; Frederiksen, T.; García, A.; Brandbyge, M. Improvements on

non-equilibrium and transport Green function techniques: The next-generation transiesta. *Computer Physics Communications* **2017**, *212*, 8 – 24.

(3) Valencia, H.; Gil, A.; Frapper, G. Trends in the adsorption of 3d transition metal atoms onto graphene and nanotube surfaces: a DFT study and molecular orbital analysis. *The Journal of Physical Chemistry C* **2010**, *114*, 14141–14153.

(4) Hoffmann, R. A chemical and theoretical way to look at bonding on surfaces. *Rev. Mod. Phys.* **1988**, *60*, 601–628.
